# Growth curves in short supply: a descriptive study of the availability and utility of growth curve data in adolescents with eating disorders

**DOI:** 10.1186/1471-2296-14-134

**Published:** 2013-09-08

**Authors:** Megan E Harrison, Nicole Obeid, Maeghan CY Fu, Mark L Norris

**Affiliations:** 1 Department of Paediatrics, Children’s Hospital of Eastern Ontario, University of Ottawa, 401 Smyth Road, Ottawa, ON, Canada; 2 Children’s Hospital of Eastern Ontario Regional Eating Disorder Program, Ottawa, ON, Canada; 3 Children’s Hospital of Eastern Ontario Research Institute, Ottawa, ON, Canada

**Keywords:** Adolescent medicine, Pediatric growth, Eating disorders

## Abstract

**Background:**

Healthy body weight (HBW) determination affects multiple aspects of eating disorder (ED) treatment. For example, it can inform patients and providers as to when return of menses (ROM), an objective determinant of health, can occur. Growth curves (GCs) are sensitive indicators of health in youth and when up to date provide critical information regarding normal and expected trajectories of growth. Although not widely recommended as a first line tool for HBW calculation, a GC guides providers selecting a HBW that is individualized to each patient. The primary aim of this paper was to assess availability and feasibility of utilizing GC data for HBW prediction in adolescents referred for an ED assessment. We also sought to determine how this calculation compared to the standardized HBW calculation that uses mean body mass index (BMI) for age and how each of these numbers compared to the actual weight at ROM.

**Methods:**

A retrospective chart review was completed on outpatients assessed for EDs between January 2004 and December 2006. A total of 102 patients met inclusion criteria. Demographic information, GC data, HBW predictions, and menstrual history were analyzed. A comparison of predicted HBWs using the aforementioned calculations and weight at ROM was performed using *t*-test analyses.

**Results:**

Eighty-one patients (79.4%) had GC data available at assessment although HBW prediction was possible in only 24 patients (23.8%) due to poor GC completion. Of those 24 patients, 9 had ROM data available; no significant difference between our predicted HBW and the weight at ROM was found in these patients. In cases where HBW predictions could be compared using GC data and the BMI method, we found the GC calculation to be overall superior.

**Conclusions:**

We found overall rates of GC completion to be very low in our patients, which in turn limited the feasibility of relying on a GC for HBW calculation in ED patients. When complete, GCs provide accurate HBWs for most patients with EDs although it is clear that secondary methods of calculation are required given the gaps in data observed using this cohort.

## Background

It is estimated that 0.5% of adolescent females in the United States suffer from anorexia nervosa (AN), 1-5% from bulimia nervosa (BN), and even more who present with subthreshold eating disorders (EDs) that do not meet exact criteria of the *Diagnostic and Statistical Manual of Mental Disorders, Fourth Edition (DSM-IV*) for AN or BN [1]. AN is a life-threatening, chronic disorder with devastating and potentially irreversible medical consequences, including cardiovascular complications, decreased bone mineral density, structural and functional brain abnormalities, and impaired growth and development [2]. Fortunately, if addressed in a timely manner, the likelihood of reversing or minimizing some of these consequences is improved. As such, prompt recognition, referral, and subsequent treatment are crucial to help minimize potential physical sequelae.

As is the case with most chronic illnesses diagnosed during childhood and adolescence, growth history is an important clinical tool in the context of ED assessment and management [3,4]. Both the World Health Organization (WHO) and the Centers for Disease Control and Prevention (CDC) encourage the use of growth charts worldwide in the routine monitoring of infants, children and adolescents [5-7]. Growth curves are sensitive indicators of health in children and adolescents that provide a standard of how children and adolescents should grow over time. Trajectories of growth are most valuable when represented as multiple points over time, as they can allow for early identification of potential health problems and provide health care providers with an earlier opportunity to screen for chronic illnesses and intervene or investigate further as required [5,6]. Growth curves also provide important information as it relates to ED diagnoses. For example, a Swedish report, which involved the retrospective analysis of school health service growth charts of young female students diagnosed with restrictive EDs, clearly showed interruptions in normal growth parameters and growth velocity in the months predating clinical diagnosis; that is, two thirds of the weight deficit (i.e. the deviation from the expected weight growth curve) and 60% of the height retardation was generated before the onset of weight loss [8]. In another smaller retrospective review of growth curves of female adolescents (mean age 13.3 years) with AN, growth arrest was again evident prior to the onset of weight loss [9].

Given the complex and multifactorial nature of AN, research into how we can best re-nourish patients is ongoing. One of the most basic questions first asked by patients and families after an assessment relates to the amount of weight gain required to re-establish health. In fact, many of our immediate clinical decisions for treatment are influenced by this “number”, that is, the patient’s weight percentage of their target/healthy body weight (HBW). If a patient is deemed dangerously low in weight (for example, if their weight was less than 70% of their expected weight), hospitalization would likely be recommended. The “number” also has implications as to the degree of medical risk associated with the early stages of refeeding. Patients initiating treatment at dangerously low weights are at increased risk of the refeeding syndrome and need to be monitored appropriately [2,10]. Thus, the manner by which we calculate a patient’s healthy weight goal is critically important not only for our immediate clinical decisions, but for patients’ psychological well-being as they attempt and begin to accept the notion of a return to a higher weight.

Despite the fact that the establishment of a HBW is a critical component in the successful management of ED patients, there is debate as to how the “number” should be best calculated [11]. Clinicians and researchers have explored different methods for calculating HBW, although recent literature points to the Body Mass Index (BMI) method as the primary method of choice when dealing with both ED and non-ED adolescent populations [4,12]. The BMI method of calculating HBW involves the use of CDC growth charts, whereby HBW is the product of the patient’s current height (in m^2^) and the 50th percentile BMI for a person of the same age and gender [4,12]. The BMI method is considered more developmentally appropriate, based on a review of 3 different methods of HBW determination (the BMI method, the McLaren method and the Moore method) in an adolescent ED population [4]. The details of the McLaren method and the Moore method are published elsewhere [12-14]. Another study compared the same 3 methods in a non-ED adolescent population [12]. The authors concluded that while all 3 methods seemed equally valid for children under the age of 8 years, the BMI method was the only one that could be consistently applied to all children aged 2 to 20 years. Another recommendation for HBW calculation has been to use a BMI percentile in the 14th to 39th percentile range, making adjustments for prior weight, pubertal stage and expected growth [3]. Despite the fact that each of these studies raises valid arguments, there are potential problems associated with these methodologies. As an example, a recent study on adolescents with AN commented on the fact that the BMI percentile ranges set out by other studies are likely to be too wide to be useful in clinical settings for individual patients, and that approximately 1 out of 5 (20%) patients are unlikely to reach reproductive maturity if such guidelines are followed [15]. Anecdotally, this makes sense understanding that in any adolescent population at a given age, there will be a percentage of patients whose HBW and BMI is lower than the 50th percentile (i.e. patients who are naturally tall and slim), and those who will need to have a BMI greater the 50th percentile to have regular menses and not be physically compromised (i.e. patients who are shorter and heavier). In addition to each of these arguments, it has also been suggested that pelvic ultrasound grading can be used as a measure of reproductive maturity, and therefore, as a means of helping to determine HBW [15]. If we consider and accept menstruation as a vital component of healthy development, then it stands to reason that this information could play a role in answering questions related to what our threshold should be for a minimal acceptable weight.

Taking all of this into consideration, completed and up-to-date growth curves provide extremely valuable information on individual growth trajectories and should be considered an important tool that can be used to help estimate a patient’s predicted HBW. We propose that in cases where an up-to-date growth curve exists, analysis of the patient’s growth curve should be the first-line measure used to provide an accurate estimate of a patient’s HBW. By using an up-to-date growth curve with multiple points documented over time, a clinician can easily establish which percentiles a patient’s weight and height were following prior to the onset of disease [16]. In most cases, it allows a clinician to easily estimate a patient’s HBW and provides a number that is specific to the individual patient based upon their previous growth parameters, rather than on the population average for age (i.e. the 50th percentile).

When accurately predicted, a HBW goal can help address patient anxiety towards weight goals early in the course of treatment by eliminating any uncertainties the patient may have with respect to the amount of expected weight gain. It also informs treatment decisions relating to the intensity of services required, and may help predict a weight required for return of menses (ROM) - an objective determinant of health in ED patients (indicative of a sufficient accrual of body fat and restoration of hormone levels to allow for the resumption of proper menstrual functioning) [16,17]. The inherent difficulty with this method lies not in the calculation, but in the reality that patients presenting for ED assessments often do so either without any documented prior growth history, or with growth charts that are incomplete or limited in the information provided.

The primary goal of this paper was therefore to explore whether the use of a growth curve provided at the time of assessment does in fact provide a feasible means of determining a HBW. We aimed to do this by 1) reviewing the availability and comprehensiveness of adolescent growth curve data of patients referred for a multidisciplinary ED assessment; and by 2) examining the relationship between our predictions of HBW goals based on prior growth curve data provided and ROM (an objective indicator of healthy weight). We also compared our proposed technique of HBW estimation, the “growth curve method”, with the BMI method of weight goal calculation to compare their accuracy.

## Methods

This study involved a retrospective chart analysis of patients who presented for an outpatient ED assessment at a Canadian tertiary care hospital over the course of a three-year period. A data abstraction sheet was created by the authors and trialed on 10% of patients to ensure feasibility. Information relating to demographics, clinical and medical variables present at assessment, diagnosis, growth curve data, and medical indicators such as menstrual status, were included in the data abstraction form. This study was reviewed and approved by the Children’s Hospital of Eastern Ontario (CHEO)’s Research Ethics Board prior to data retrieval. Data was tabulated using SPSS 17.0 and descriptive, frequency, and *t*-test analyses were performed where appropriate.

### Participants

Participants consisted of all patients who presented to the tertiary care facility for an outpatient ED assessment between January 2004 and December 2006. In total, 115 individuals were identified.

### Measures

#### Demographic information

Variables such as age at assessment, age of self-reported ED onset (i.e. onset of ED behaviours such as calorie restriction, over-exercising), diagnosis at assessment, and BMI (kg/m^2^), were noted.

#### Growth curve data

Abstracted data relating to growth curve information included whether prediction of healthy body indices was performed at assessment, whether growth curves were available before and after assessment, who provided the growth curve data, how many data points were available before and after ED onset, and whether there was evidence of growth retardation, as indicated by any significant deviance away from the patients’ expected growth curve based on his/her historical growth curve data.

#### Healthy Body Weight Predictions

HBW predictions were performed by reviewing historical growth curve data prior to ED onset and noting the percentile curves that patients’ weight and height were previously following. We then extrapolated this to the weight at that same percentile for current age. We also extrapolated the patient’s previous BMI curve percentile to the same percentile for current age. In our practice, this HBW goal is the minimum number of a 2–3 kilogram weight range that is set as the goal range. For the purpose of this paper, we used this minimum target weight to compare to weight at ROM. HBW predictions were also made using the BMI method of calculating HBW (i.e. using the 50th percentile BMI for exact age and height on the CDC BMI-for-age percentile charts) in order to compare the results of our proposed technique and this established method.

## Results

### Demographic and growth chart data

There were 115 patients assessed during the study timeframe. Of those, 13 were excluded for various reasons: 8 patients (7.0%) were assessed on an emergency/inpatient basis and therefore standard outpatient assessment data was not obtained, 1 patient (0.9%) had no assessment data available, and 4 patients (3.5%) were found to have no ED diagnosis after assessment. This left a total of 102 patients for analysis and review. The majority (n = 94; 92%) of patients reviewed were female, and the mean age of the sample was 15.50 years (*SD* = 1.47). Twenty-nine patients (28.4%) were diagnosed with AN, 23 patients (22.5%) were diagnosed with BN, and the remaining 50 patients (49%) were given a diagnosis of eating disorder not otherwise specified (EDNOS). Table 1 contains pertinent demographic characteristics of this sample separated by gender.

**Table 1:** Means (SD) of gender-specific demographic characteristics described per diagnostic category

**Demographics**	**Females**			**Males**		
	**AN (n = 25)**	**BN (n = 23)**	**EDNOS (n = 46)**	**AN (n = 4)**	**BN (n = 0)**	**EDNOS (n = 4)**
Age of patient at assessment (yrs)	15.06 (1.76)	16.49 (1.08)	15.41 (1.29)	15.06 (1.01)	--	13.98 (.87)
Age of patient at ED onset (yrs)	14.02 (1.73)	14.16 (1.72)	13.40 (1.62)	14.14 (1.22)	--	13.35 (.96)
Chronicity of symptoms (yrs)	1.04 (.68)	2.33 (1.44)	2.01 (1.30)	.92 (.22)	--	.62 (.26)
Height at assessment (meters)	1.65 (.10)	1.64 (.06)	1.64 (.07)	1.65 (.07)	--	1.64 (.10)
Weight at assessment (kg)	43.56 (7.92)	57.30 (7.60)	52.07 (8.02)	46.10 (5.36)	--	49.10 (7.11)
BMI at assessment (kg/m^2^)	15.84 (1.55)	21.41 (2.56)	19.39 (2.78)	16.87 (1.77)	--	18.20 (.71)
BMI-SDS at assessment (kg/m^2^)	−2.07 (.98)	.09 (.76)	-.45 (1.04)	−1.61 (1.44)	--	-.43 (.41)

**Figure 1:**
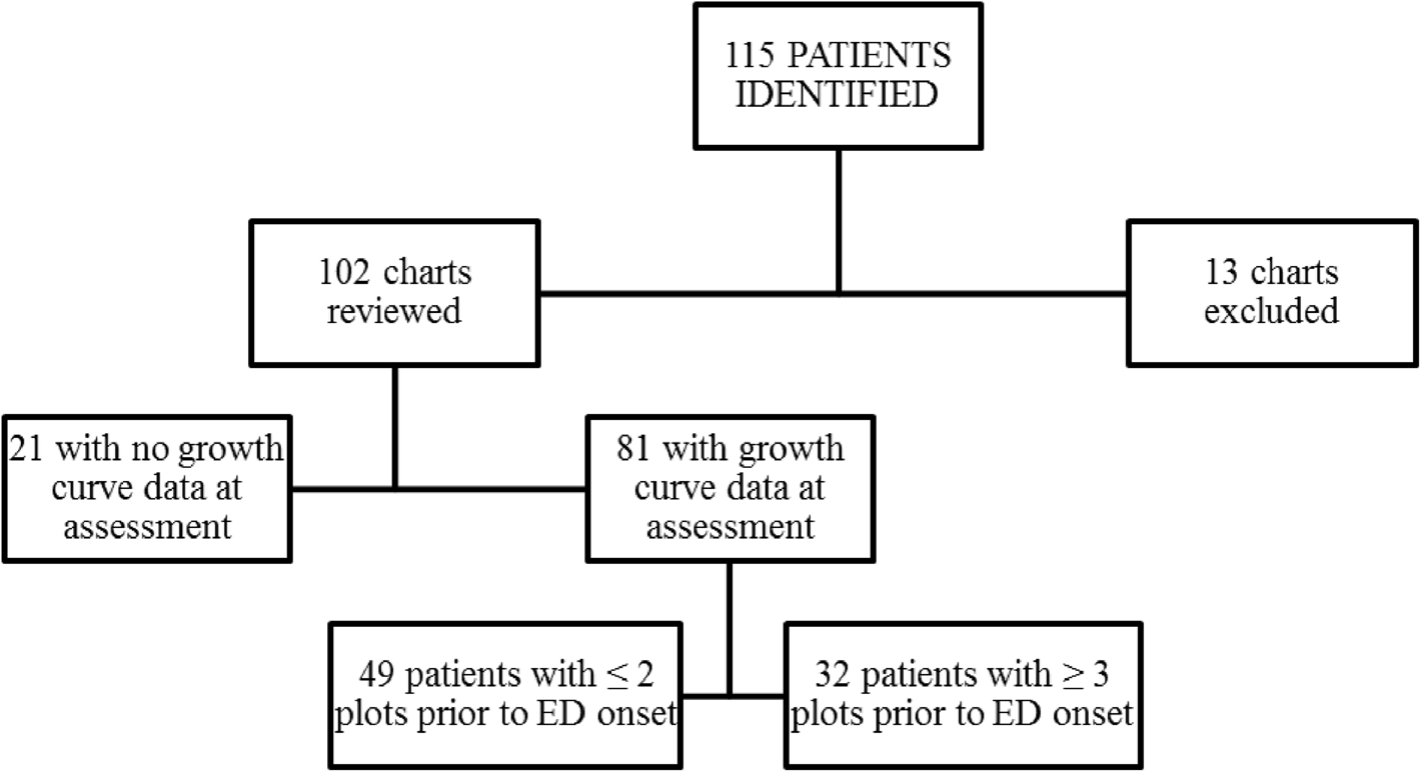
Growth curve availability.

Most of the data available for growth curves was provided by the patients’ primary care physician (86.6%), although specialists (4.9%), family members (6.1%), and self-reported data (2.4%), were also included. Eighty-one participants (79.4%) had at least one growth curve weight data point available for analysis (regardless of whether the data was captured before or during the onset of the ED), although 60% of all growth curves reviewed contained 2 or fewer weight points at the time of assessment (weight median 2.0, range 0 – 47). Clinically, this would represent at most two weight points on a growth curve, one of which would likely have been completed as part of the referral process. When available, patient weights were documented by primary health care providers on average 11.93 months (*SD* = 13.07 months) after self-reported ED onset. Figure 1 shows the patient growth curve availability.

### Using growth curves for estimation of HBW goals

**Figure 2:**
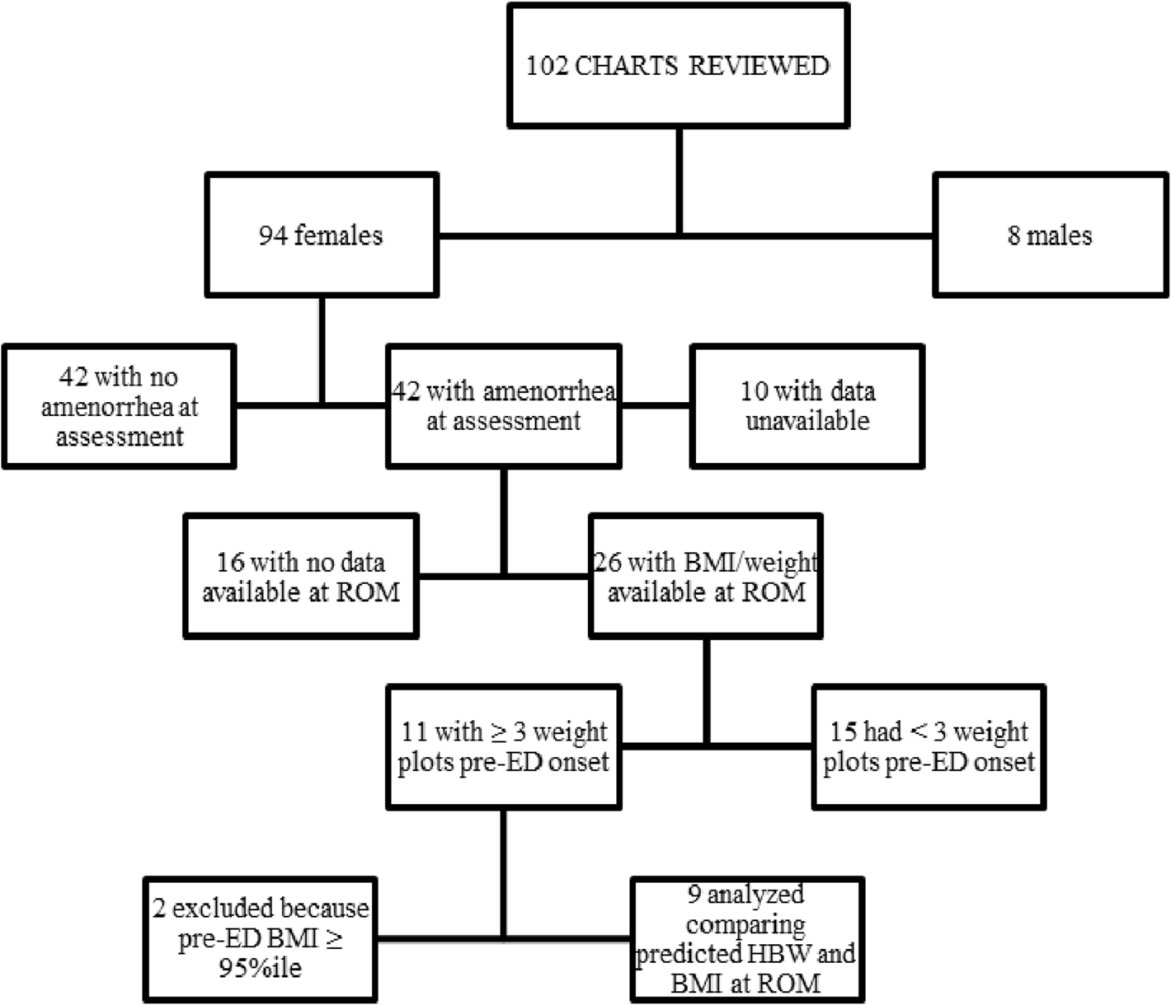
Flowchart of patient selection for comparison of predicted HBW at assessment and BMI at ROM.

In an attempt to estimate how accurate the predicted HBW was at the time of assessment, we compared the HBW estimations provided at assessment to weights at time of ROM in patients where data was available. Of the female patients diagnosed with either AN or low-weight EDNOS at assessment, only 26 patients had secondary amenorrhea, were not taking any form of contraceptive throughout the treatment course, and had available weight data at the time ROM occurred. Of these, only 11 patients (42%) had more than 2 weight plots on their growth curve prior to the onset of the ED. Two of these patients were excluded from further analysis as their pre-ED BMI and weights were greater than the 95th percentile, making it extremely difficult to predict a HBW trajectory based upon ROM only. This left just 9 patients who had appropriate pre-ED growth curve data for an estimation of a HBW goal. Figure 2 explains patient selection for comparison of predicted HBW at assessment and weight at ROM. The results of the *t*-test did not show any significant difference between the predicted HBW at assessment (*M* = 55.98 kg, *SD* = 4.63) and the weight at ROM (*M* = 56.04 kg, *SD* = 4.22) (*t*(8) = −0.094, *p* = .927).

Results show that 8 (89%) patients experienced ROM within 2 kg of the target weight set at assessment, while the remaining patient had an estimation within 5 kg of her weight at ROM. Of note, in the single outlier, her growth curve did not contain any weight information for the 6 years preceding the onset of illness. Each patient whose predicted HBW was within 2 kg of their weight at ROM had at least one weight plot within 1 to 2 years prior to ED onset.

**Figure 3:**
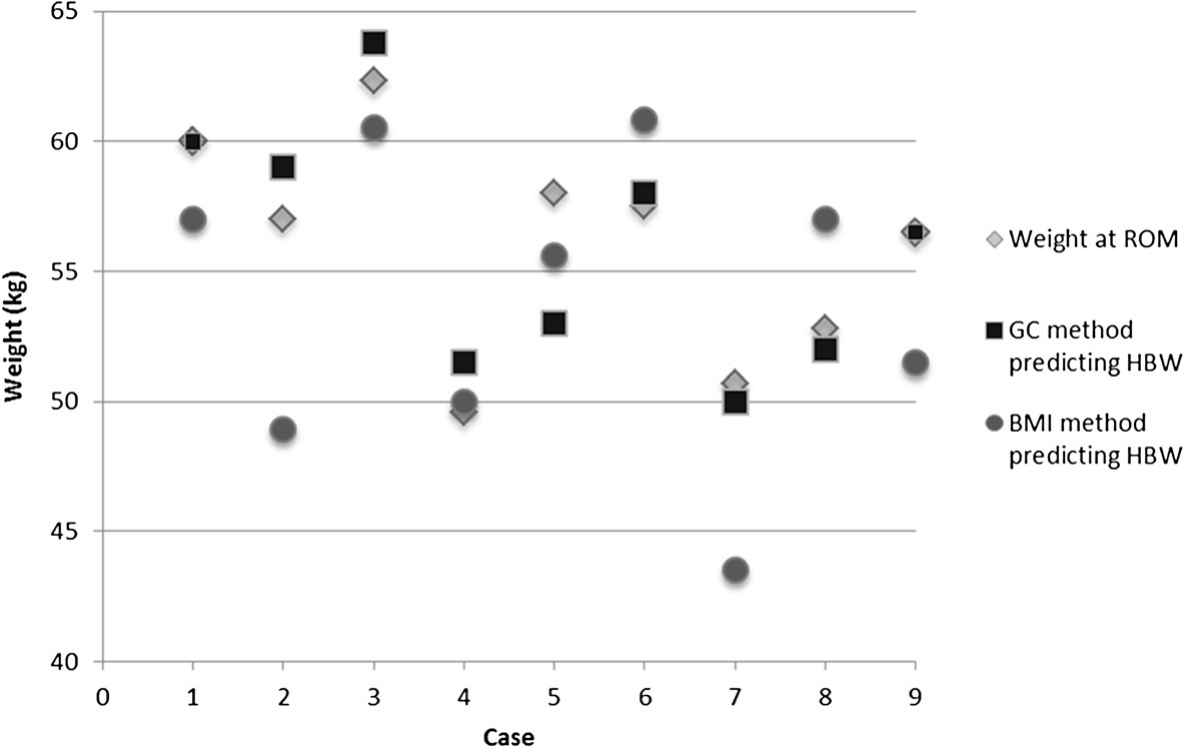
Differences between weight at return of menses (ROM) in comparison to the growth curve (GC) and BMI method of healthy body weight (HBW) prediction.

A comparison between the growth curve method and the BMI method is shown in Table 2. Overall, a descriptive look at the table demonstrates that the growth curve method generally provides a HBW estimation that is closer to the weight at ROM than does the BMI method. This can be seen when reviewing the difference scores between the two methods. On average, the growth curve method generated HBWs that were 0.067 kg greater than the weight at ROM, whereas the BMI method generated HBWs that were 2.178 kg greater than the weight at ROM. Also interesting is the range of scores that emerged using the two different methods. With the growth curve method, the predictions ranged from being under-estimated by 2 kg to an overestimation of 5 kg. With the BMI method, the predictions range from being underestimated by 4.2 kg to an overestimation of 8.1 kg. Although these comparisons are only available for a small number of individuals (n = 9), thereby affecting the ability to perform statistically meaningful comparisons across the methods, this provides some preliminary descriptive data on the accuracy of HBW predictions using the different methods. The comparison of the growth curve method and BMI method of weight goal predictions as they relate to ROM is depicted in Figure 3.

**Table 2:** Assessment and predicted weight characteristics, and the difference scores between methods of prediction

**Case**	**Assessment**				**Predicted HBW using different methods (kg)**		**Return of menses (ROM; kg)**	**Difference between HBW predictions (kg)**	
	**Weight (kg)**	**Height (cm)**	**BMI (kg/m**^**2**^**)**	**BMI-SDS (kg/m**^**2**^**)**	**Growth Curve (GC) method**	**BMI method**		**GC method – ROM**	**BMI method – ROM**
**1**	49.9	165.4	18.2	−0.99	60.0	57.0	60.0	.00	3.00
**2**	40.7	160.0	15.7	−1.54	59.0	48.9	57.0	−2.00	8.10
**3**	46.0	172.8	15.4	−2.63	63.8	60.5	62.3	−1.50	1.80
**4**	46.9	162.3	17.8	−0.04	51.5	50.0	49.6	−1.90	-.40
**5**	46.0	167.0	16.5	−1.63	53.0	55.6	58.0	5.00	2.40
**6**	57.1	172.0	19.3	−0.46	58.0	60.8	57.5	-.50	−3.30
**7**	39.4	149.0	15.6	−1.23	50.0	43.5	50.7	.70	7.20
**8**	40.8	165.0	15.1	−3.48	52.0	57.0	52.8	.80	−4.20
**9**	37.3	166.0	13.5	−2.82	56.5	51.5	56.5	.00	5.00
**Average**	**44.9**	**164.4**	**16.3**	**−1.65**	**55.98**	**53.89**	**56.04**	**.067**	**2.18**

## Discussion

Our data suggests that the majority of patients referred for an ED assessment have incomplete growth curves. Despite a mean age of 15.5 yrs at assessment, the majority of patients presented with 2 or less growth curve weight plots prior to ED onset, suggesting either a low number of medical doctor (MD) visits during the childhood and adolescent years and/or incomplete data collection at the time of the MD visit. Literature reviewing how often patient growth charts are kept up-to-date is sparse. Two studies performed in the pediatric inpatient setting found that rates of documentation of growth parameters in the hospital were suboptimal [18,19]. In the outpatient primary care setting, it has been shown that many practitioners report not measuring children at every health visit and when measured, the data is often not plotted or analyzed on growth curves [20,21]. This is concerning because of the potential delay in the diagnosis of a variety of illnesses that impair growth. Moreover, our observation that patient weights were only documented, on average, approximately one year after self-reported ED onset further highlights the importance of annual health screenings.

Although the sample of patients with sufficient growth curve data deemed necessary to calculate an estimated target weight was very small in our study (35%), we were able to demonstrate good utility when completed and up-to-date growth curves were available for patients with AN/restrictive EDNOS. Although we cannot comment definitively based upon our low sample size, it makes clinical sense that the sensitivity of such predictions would only improve when growth curves contain more data points for height and weight and are completed on an annual basis.

Unfortunately, there is no current consensus among providers regarding an ideal method of HBW calculation in patients with EDs [11]. Research into this area, while limited, has explored the utility of the BMI, McLaren, and Moore methods of HBW calculation, as well as other methods such as pelvic ultrasound grading, in both ED and non-ED adolescent and adult populations [3,4,12,15]. While no clear consensus has been established, the BMI method (i.e. the use of BMI growth curves) is starting to emerge as a primary choice among researchers for HBW calculations in clinical and non-clinical children and adolescents [4,12]. In this study, we used ROM as an objective indicator of weight restoration, although many would argue that this alone may be insufficient. Of the studies that have been completed as a means of addressing this question, most have shown that menses returns at an average weight around 90% of an estimated HBW [22-25]. It is important to note, however, that the means by which the HBW was calculated in these studies varied, although authors typically did so using the median weight for height and/or age as the reference point for calculation. Given the number of confounders associated with this issue, it is not surprising that the level of individual variation is considerable. For example, several authors have shown that anywhere from 5% to over 30% of patients remain amenorrheic once they reach 90% of their estimated “normal” body weight [22-25]. In one study, 48% of patients who resumed menses did so at a weight less than 90% of the “standard body weight” (range 75 to 115%) [23]. Another study showed that ROM occurred at a weight above the population average in 31% of patients [16]. Clearly, further research into this area is required. To our knowledge, there are no other publications using historical growth curve data as a means of estimating HBW, nor comparing it to HBW based on ROM. We were also unable to find any published reference on how calculation differences (i.e. using growth curves alone vs. standardized calculation of percentile of BMI) influence rates at which ROM is demonstrated. It is also important to note that it is unclear how close the correlation between BMI and ROM is expected to be. There is a complex relationship between leptin, adiponectin, inhibin B, ghrelin and a disrupted hypothalamic regulation of menstruation that likely effects the direct correlation between BMI and ROM [26].

We believe that our data, despite its limitations, shows obvious merit in the argument that growth curves should be used as a first line to predict HBW whenever sufficient data is available and allows for accurate prediction. We have shown in our small sample that using historical growth curve data to predict HBW goals is more accurate than using the BMI method of calculation. Although a small sample, the implications for the individual patients are note-worthy. For example, the HBW prediction done by the BMI method for one of our patients was 7.2 kg lower than the weight required for ROM, and 0.7 kg lower when done by the growth curve method. Another patient’s predicted HBW goal done by the BMI method was 8.1 kg below the weight required for ROM, as compared to 2 kg above the target when done by growth curve method. Clinically, overshooting and undershooting these weight goals for patients can lead to distrust of the treatment team and plan, as well as unneeded patient anxiety. As such, we recommend that ED clinicians make every possible effort to gather prior growth information at the time a first assessment is completed. Our own program has made it mandatory for all referred patients to have a completed growth curve sent in at the time of referral, although it is clear, based upon this study, that the majority of providers are not collecting such data longitudinally. We also recommend clinicians use any other means available, such as reviewing old hospital charts, triage visit records from emergency department visits, as well as having the family provide data whenever possible. Only after this method has been exhausted and/or deemed not applicable should we look at other methods of calculating HBWs. It is important to note that in growing and developing youth, HBW goals need to be continually adjusted as height increases, to maintain the same goal BMI percentile. Using historical growth curve data is of course limited to having data available, and our study has shown that growth curves in this population are often not complete. In those cases, the BMI method of HBW calculation can be used. Another noted limitation to our method involves HBW predictions for patients who were overweight prior to ED onset. We removed 2 patients from analysis because their pre-ED BMIs were greater than the 95 percentile for age. In those cases, weight trajectory as seen on growth curves are of limited use with our method of HBW goal prediction. In those cases as well, perhaps the BMI method of calculation is most appropriate. Further research is required exploring accurate methods of estimating HBW for those who were overweight prior to ED onset, as well as for males and pre-menarchal females.

It should be stated that, as is typically the case with pediatric ED populations [27], the observed proportion of EDNOS patients in this study is much greater than that of both the AN and BN patients, although this does not limit the representativeness of the findings. A diagnosis of EDNOS is given to patients who do not exactly meet the strict criteria set forth by the DSM-IV for a diagnosis of either AN or BN, and it is this diagnostic category that predominates the treatment-seeking adolescent ED population [27]. Additionally, for the purposes of this study, the population of interest consisted of patients with AN and those with restrictive EDNOS, as the determination of a “healthy weight” is most relevant for these patients at the time of assessment.

Limitations of our study include the fact that it was retrospective in nature and relied upon chart review. As noted above, the use of ROM as the primary indicator of heath is limited for a variety of different reasons, although this alone would suggest that patients have regained sufficient fat stores and hormone levels to allow menstrual functioning to resume. Although used as a marker of health in ED patients in this study, ROM should be regarded as the first step required for maintenance of medical health, as one of the primary goals of treatment is to re-establish regular ovulatory menstrual cycles [16]. We were also limited in our analysis of the reliability of our predictions due to the low number of growth curves completed with sufficient data points. Non-significant results may also have been found due to the small sample size available for this study.

## Conclusions

Adolescents presenting with EDs have limited growth curve data available at the time of assessment, which makes it challenging to uniformly utilize this tool to estimate a HBW goal. Despite this, the use of growth curves to estimate HBW in adolescents with AN or restrictive EDNOS should be considered whenever possible. When this method is not possible (i.e. due to incomplete growth charts), it becomes important to then use an alternative method (e.g. the 50th percentile BMI method) that has shown some clinical merit. In these cases, clinicians should be aware of the limitations of the selected method and use other clinical indicators such as ROM to help guide final decisions.

This study also suggests that we need to put greater emphasis on anticipatory screening and the importance of annual health checks for all youth. At those visits, health care practitioners should make a point of always collecting weight and height data as a means of facilitating the completion of growth curves. In addition, the public should continue to be educated regarding the merit of anticipatory guidance and regular annual medical visits with primary care providers across all ages, in order to ensure all developmental and physical milestones are being met. More prospective research is needed regarding an optimal means of calculating HBW goals for children and adolescents with EDs.

## Abbreviations

AN: Anorexia nervosa; BN: Bulimia nervosa; ED: Eating disorder; DSM-IV: Diagnostic and statistical manual of mental disorders, fourth edition; WHO: World Health Organization; CDC: Centers for disease control and prevention; HBW: Healthy body weight; ROM: Return of menses; BMI: Body Mass Index; EDNOS: Eating disorder not otherwise specified.

## Competing interests

The authors declare that they have no competing interests.

## Authors’ contributions

MH participated in the design of the study, completed the chart review and data acquisition, and drafted the manuscript. NO participated in the design of the study, performed the statistical analysis, and participated in drafting the manuscript. MF was involved in drafting the manuscript. MN conceived the study, participated in its design and coordination, and helped to draft the manuscript. All authors read and approved the final manuscript.

## Pre-publication history

The pre-publication history for this paper can be accessed here:

http://www.biomedcentral.com/1471-2296/14/134/prepub
